# Temporal patterns of prefrontal hemodynamics during Tai Chi, Baduanjin, and Liuzijue: a wearable fNIRS study protocol

**DOI:** 10.3389/fpsyg.2026.1924514

**Published:** 2026-10-14

**Authors:** Yunlong Pang, Qun Fang

**Affiliations:** College of Physical Education, Qingdao University, Qingdao, China

**Keywords:** Baduanjin, fNIRS, Liuzijue, prefrontal oxygenation, Tai Chi, traditional mind–body practices

## Abstract

Traditional mind–body practices integrate movement, breathing, and attention, but the temporal characteristics of prefrontal hemodynamics during ongoing practice remain unclear. This study protocol aims to compare process-level patterns of prefrontal oxygenation during 24-form Tai Chi, Baduanjin, and Liuzijue. Approximately 45 healthy college students aged 18–22 years are expected to be enrolled. Recruitment will continue, if necessary, until 36 participants have provided complete and analyzable data for all three conditions. In a within-participant Latin-square design, each participant will complete the three practices on separate days. Each session will include a 120-s quiet-standing baseline followed by one 6-min practice trial with standardized audio cues. Wearable functional near-infrared spectroscopy will continuously record HbO and HbR over the bilateral dorsolateral and frontopolar prefrontal cortex, alongside synchronized respiratory, photoplethysmographic, and motion signals. Temporal changes will be characterized using overlapping 30-s windows advanced in 15-s steps. The primary outcome will be the overall 6-min linear slope of global prefrontal HbO. Differences among practices will be examined using a linear mixed-effects model adjusted for session period and sequence; models additionally incorporating respiratory, cardiac, and motion measures will be treated as sensitivity analyses. Secondary time-resolved and region-specific outcomes will be exploratory. This protocol provides a prespecified method for comparing fNIRS-derived prefrontal hemodynamic patterns during traditional mind–body practices, which may include residual systemic components.

## 1 Introduction

Traditional Chinese mind–body practices are important components of Chinese culture, yet their prefrontal hemodynamic characteristics remain insufficiently understood. Core concepts such as intention-guided movement, breath regulation, mind–body coordination, and internal cultivation have developed mainly from experiential training and traditional cultural contexts (Larkey et al., 2009; Yeung et al., 2018; Shi et al., 2020). Selected components can nevertheless be translated into measurable scientific questions. Intention-guided movement may be examined through goal-directed action and sustained attention, breath regulation through respiratory timing and movement coordination, and mind–body coordination through the integration of movement, breathing, and attentional focus (Larkey et al., 2009; Friedman and Robbins, 2022). Such investigation does not validate broad traditional concepts such as internal cultivation, but may provide empirical evidence for some observable processes and strengthen the scientific communication of research on traditional mind–body practices.

Tai Chi, Baduanjin, and Liuzijue are slow and stable traditional mind–body practices suitable for process-based observation. Tai Chi involves continuous multi-joint sequences, weight shifting, and postural transitions; Baduanjin comprises eight standardized movement units coordinated with breathing; and Liuzijue emphasizes controlled exhalation and vocalization accompanied by simple movements (Henz and Schöllhorn, 2018; Shi et al., 2020; Chen et al., 2022). Their differences in movement complexity, respiratory involvement, and attentional demands provide a comparative model for examining whether these characteristics are accompanied by distinguishable temporal patterns of prefrontal oxygenation. Because direct comparative evidence is limited, this study is exploratory and does not specify directional hypotheses for HbO or HbR.

The prefrontal cortex (PFC) was selected because it supports cognitive control and executive functions, including sustained attention, working memory, goal-directed behavior, and adjustment to task demands (Friedman and Robbins, 2022). These functions are relevant when practitioners execute movement sequences, monitor posture, coordinate breathing, and maintain attentional focus. Wearable functional near-infrared spectroscopy (fNIRS) permits continuous recording of cortical hemodynamic signals during relatively natural movement and is therefore suitable for examining process-level changes (Herold et al., 2018; Yücel et al., 2021). Previous fNIRS studies have measured prefrontal responses during Tai Chi, dual-task gait, walking, cycling, and exercise–cognition tasks, supporting its feasibility in movement settings (Belluscio et al., 2021; De Wachter et al., 2021; Robinson et al., 2021; Chen et al., 2022; Qi et al., 2024; Shen et al., 2024; Wang et al., 2025). However, fNIRS measures hemodynamic rather than direct neural electrical activity, and PFC recording cannot represent the complete neural network involved in these practices.

Studies of Tai Chi, Baduanjin, and Liuzijue have mainly examined psychological, physiological, and health-related outcomes through interventions, pre–post comparisons, or subsequent cognitive and dual-task assessments (Yang et al., 2022; Zhang et al., 2023; Zhang and Jiang, 2023; Feng et al., 2025). These designs provide limited information about how prefrontal oxygenation changes throughout a complete routine. Its temporal variability and possible stabilization during practice remain unclear, and direct within-subject comparisons of the three practices are scarce.

This protocol therefore aims to compare process-level prefrontal oxygenation during Tai Chi (24-form), Baduanjin, and Liuzijue. Using a within-subject design and continuous wearable fNIRS recording, it will characterize temporal trajectories, variability, and exploratory stabilization features of HbO and HbR. The study extends the existing research on traditional mind–body practices from outcome-based evaluation to process-based description and provides a methodological basis to investigate observable aspects of traditional practice concepts. The findings may inform future research on the prefrontal hemodynamic patterns associated with these practices. However, the present study does not directly test broad traditional concepts such as “internal cultivation.”

## 2 Methods and analysis

### 2.1 Participants

#### 2.1.1 Recruitment and sample-size determination

Using purposive sampling, healthy college students aged 18–22 years at Qingdao University will be recruited until 36 participants have provided complete and analyzable fNIRS, respiratory, PPG, and motion data for all three practice conditions. Approximately 45 participants are expected to be enrolled, but this is a planning estimate rather than a fixed maximum. The age range of 18–22 years was selected to correspond to the target undergraduate population and to maintain a relatively homogeneous young-adult sample.

An *a priori* power analysis was conducted using G*Power 3.1.9.7 with the “ANOVA: Repeated measures, within factors” procedure, specifying one group and three measurements. Because no directly applicable pilot estimate was available, the calculation assumed Cohen’s conventional medium effect size (*f* = 0.25), an *α* level of 0.05, statistical power of 0.90, a correlation of 0.50 among repeated measurements, and a nonsphericity correction of 1.00. The analysis indicated that 36 complete datasets were required (Cohen, 1988; Faul et al., 2007). The anticipated enrollment of approximately 45 participants allows for approximately 20% loss due to withdrawal, failure to meet the performance-competence criteria, or unusable fNIRS or auxiliary-sensor data. Recruitment will continue beyond 45 participants if necessary and will stop only when 36 complete and analyzable datasets have been obtained. This calculation supports the omnibus comparison of the primary outcome—the overall 6-min linear slope of global PFC HbO—across the three practice conditions. The repeated-measures ANOVA calculation was used as an approximation for the planned random-intercept linear mixed-effects model (LMM). Because the primary analysis requires complete data for all three conditions and the random-intercept model implies compound symmetry, the calculation addresses the same 2-df within-participant omnibus practice comparison under its stated assumptions. However, session period and assigned Latin-square sequence are not represented explicitly in G*Power. Thus, 36 complete datasets is a pragmatic target rather than an exact LMM-derived requirement, and departures from compound symmetry could reduce power. The time-resolved and ROI-specific analyses were not separately powered and will remain exploratory.

#### 2.1.2 Eligibility criteria

Participants who meet the following inclusion criteria will be recruited: (a) students aged 18–22 years who meet the performance-competence criteria described below for 24-form Tai Chi, Baduanjin, and Liuzijue after standardized training; (b) no history of neurological disease, major physical illness, or diagnosed mental disorder; (c) no use of medications that may affect the central nervous system or hemodynamic responses within the past 2 weeks; and (d) no scalp wounds or other head conditions that preclude reliable placement of the fNIRS probes.

On the other hand, participants will not be considered for participation for any of the following reasons: (a) sleep deprivation or physical discomfort on the test day that interferes with completion of a practice session; and (b) failure to meet the performance-competence criterion after additional training and reassessment.

#### 2.1.3 Performance-competence assessment

After completing the basic standardized training described in Section 2.2.2, each participant will complete two full 6-min trials of each practice using the same standardized audio cues as in the formal experiment. The instructor will assess each trial using a prespecified pass/fail checklist. Performance competence will be confirmed only when the participant meets all of the following criteria in two consecutive trials: (a) completes all 24 forms of Tai Chi, eight sections of Baduanjin, and six exercises of Liuzijue in the prescribed sequence; (b) completes the practice continuously without omitted or repeated sections or interruption; (c) follows the standardized audio cues for movement transitions, rhythm, and the required movement–breathing coordination; (d) produces the six Liuzijue vocalizations in the prescribed sequence; and (e) requires no demonstration or additional verbal or physical prompting. Participants who do not initially meet these criteria will receive additional training and be reassessed as described in Section 2.2.2. Those who remain unable to meet the criteria after the maximum permitted training will be excluded.

#### 2.1.4 Participant characteristics and reporting

Sex, age, previous experience with each practice, and habitual physical activity assessed using the International Physical Activity Questionnaire–Short Form (IPAQ-SF) will be recorded (Craig et al., 2003). Mean age, standard deviation, and range; sex distribution; previous experience with each practice; habitual physical activity; and the number of participants included in the final analysis will be reported. For each practice, the actual number of training sessions and cumulative training duration will also be reported, together with the numbers of participants who passed the initial competence assessment, passed after additional training and reassessment, or were excluded because they did not meet the competence criteria.

### 2.2 Experimental design

#### 2.2.1 Study design and practice conditions

This study will use a within-subject design, with the order of the three practices counterbalanced using a Latin square. Each participant will complete one formal trial of 24-form Tai Chi, Baduanjin, and Liuzijue on three separate testing days.

These practices were selected because they represent different movement–breathing–attention structures among traditional mind–body exercise systems. The 24-form Tai Chi routine and the 6-min versions of Baduanjin and Liuzijue will each be standardized to exactly 360 s, providing equal observation periods for comparing temporal changes in prefrontal oxygenation. The selected versions retain all 24 forms, eight sections, and six exercises, respectively, in their prescribed sequence. For each practice, a fixed 360-s audio track containing the same low-volume non-lyrical instrumental background and practice-specific verbal cues will be used during both training and formal testing. The cue content, timing, movement sequence, breathing instructions, and Liuzijue vocalization schedule are prespecified in Supplementary Table S1. The same audio volume will be used for all participants and conditions, and no additional verbal instruction will be provided during data collection. The analysis interval will extend from the routine-onset marker to the routine-offset marker and will contain exactly 360 s of data.

In these practices, head movements are generally limited, and sudden large-amplitude movements are uncommon. Nevertheless, body movement, breathing, and vocalization may still affect fNIRS signals and will be monitored and addressed as described below.

No non-practice control condition will be included because the study aims to compare prefrontal oxygenation patterns among the three active practices. Therefore, the findings will indicate differences in fNIRS-derived prefrontal hemodynamic patterns among the traditional mind–body practices, which may include residual systemic components.

#### 2.2.2 Standardized training

Before data collection, each participant will complete five 40-min standardized training sessions for each practice over a three-week period, resulting in 15 sessions in total and 200 min of minimum training for each practice. All sessions will be delivered by the same instructor using the fixed 360-s audio tracks employed in the formal experiment. Each session will include instruction in the prescribed movement sequence and practice-specific technical requirements, repeated practice with the standardized audio, and feedback on movement–breathing coordination, rhythm control, and responses to the standardized cues. Previous task-based fNIRS studies have similarly provided pre-experimental Tai Chi training to ensure that participants can complete the required motor tasks during data collection (Xie et al., 2019; Zhang et al., 2024). Standardized task familiarization is also consistent with methodological recommendations for reducing learning-related variability during exercise-based fNIRS assessment (Herold et al., 2018).

The five sessions for each practice will constitute a common minimum training dose, but eligibility for formal testing will be determined by performance competence rather than attendance alone. Participants who do not meet the competence criteria for a particular practice will receive an additional 40-min training session for that practice, followed by reassessment. Additional training and reassessment will continue until the participant meets the criteria, with a maximum of eight training sessions permitted for each practice. Participants who remain unable to meet the criteria for any of the three practices after the maximum number of sessions will be excluded.

#### 2.2.3 Test schedule and procedure

The three formal test sessions will be scheduled on separate days with at least 24 h between sessions. Participants will be asked to avoid strenuous exercise, caffeine-containing drinks, and alcohol before each test session, consistent with previous exercise-related fNIRS protocols designed to reduce physiological confounding effects (Robinson et al., 2021). Compliance with these requirements and the participant’s sleep and physical condition will be confirmed before each session.

Before the quiet-standing baseline, participants will complete a 7-min resting-state recording while seated comfortably with their eyes open and fixed on a centrally positioned cross. They will be instructed to remain still and awake (Wang et al., 2017). After the recording equipment has been fitted and signal quality confirmed, participants will complete a 120-s quiet-standing baseline. They will stand upright with their feet approximately shoulder-width apart and arms relaxed, keep their eyes open and fixed on a mark at eye level, remain silent, and avoid unnecessary movement. After signal stabilization, the mean of the final 30 s will serve as the pre-practice reference for baseline correction (Herold et al., 2018; Robinson et al., 2021).

Participants will then perform the assigned routine continuously for 6 min using only the standardized audio track. fNIRS and synchronized respiratory, photoplethysmography, and motion signals will be recorded continuously and without interruption. The beginning and end of the routine and the start of each form, posture, or movement unit will be marked to align the physiological signals with the practice process. Immediately after the 6-min practice, participants will return to the seated position and complete a second 7-min resting-state recording under the same conditions. The two recordings will be used to explore acute practice-associated changes in prefrontal functional connectivity.

### 2.3 Data collection

#### 2.3.1 fNIRS acquisition and montage

During each formal test, prefrontal hemodynamic signals will be recorded continuously using a wearable continuous-wave NIRSport2 fNIRS system (NIRx Medical Technologies, Berlin, Germany). The system will be configured with eight dual-wavelength light sources, seven standard detectors, and one short-separation detector bundle. The standard optodes will form 20 long-separation channels with source–detector distances of approximately 30 mm, covering the bilateral dorsolateral prefrontal cortex (DLPFC) and frontopolar prefrontal cortex. The short-separation bundle will form eight 8-mm channels, with one short-separation detector adjacent to each source, to record superficial hemodynamic signals. Short-separation channels are included because long-separation fNIRS signals contain both cortical and extracerebral components (Yücel et al., 2015; Wyser et al., 2020; Yücel et al., 2021).

The system will operate at wavelengths of 760 and 850 nm with a sampling rate of 10 Hz. Recording will begin before the pre-practice resting-state recording and continue without interruption until completion of the post-practice resting-state recording. The source–detector layout and channel numbers will be presented in Figure 1, and the template-based candidate channel-to-region-of-interest (ROI) mapping and prespecified anatomical eligibility criteria will be provided in Supplementary Table S2.

**Figure 1:**
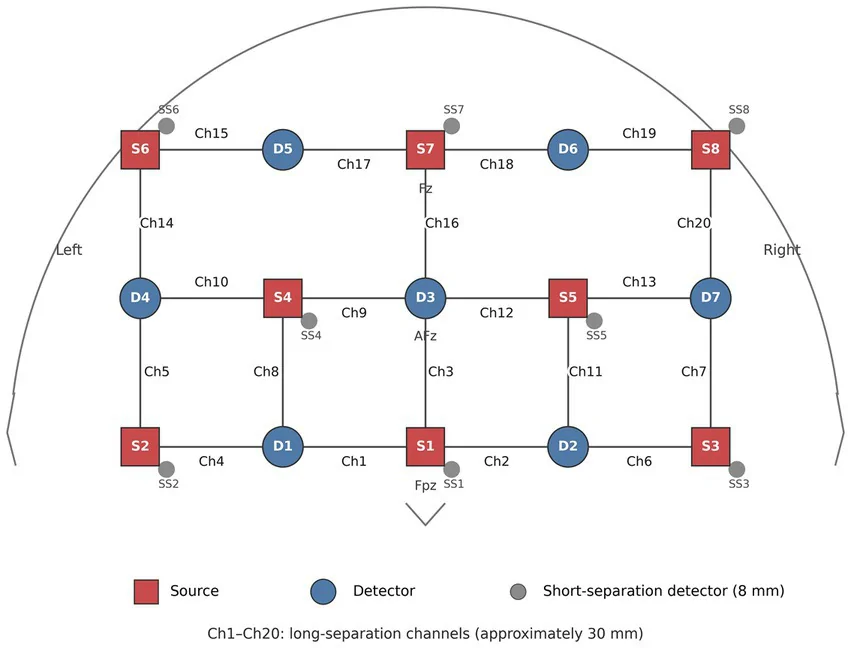
Schematic arrangement of the prefrontal fNIRS montage. Eight light sources (red squares) and seven standard detectors (blue circles) form 20 long-separation channels with source–detector distances of approximately 30 mm. One bundle of eight short-separation detector probes (gray circles) forms eight 8-mm channels adjacent to the corresponding sources. The montage is aligned using Fpz, AFz, and Fz as reference positions and is designed to cover the bilateral dorsolateral and frontopolar prefrontal cortices. The template-based candidate channel-to-ROI mapping and prespecified anatomical eligibility criteria are provided in Supplementary Table S2; final session-specific channel inclusion will be determined using three-dimensional digitization and AtlasViewer-derived cortical sensitivity profiles before examination of the fNIRS outcomes. The montage topology was adapted from von Au et al. (2023).

#### 2.3.2 Optode registration and signal quality

The montage will be positioned according to the international 10–10 system using the nasion, inion, left and right preauricular points, Fpz, and Cz as anatomical references (Jurcak et al., 2007). At each session, head circumference and nasion–inion and left–right preauricular distances will be measured; Cz will be identified from the intersection of the two arcs, and the cap will be aligned along the midsagittal plane. After cap placement, the three-dimensional coordinates of the nasion, inion, left and right preauricular points, Cz, and the center of every source and detector holder will be recorded using a FASTRAK electromagnetic digitizer (Polhemus, Colchester, VT, USA), as recommended for reproducible fNIRS optode registration (Yücel et al., 2021). Session-specific coordinate files will be imported into AtlasViewer and registered to a standard adult head model to estimate channel sensitivity profiles, Montreal Neurological Institute coordinates, and anatomical correspondence with the prespecified ROIs (Aasted et al., 2015). The same cap size and montage will be used for each participant across all three sessions. After landmark-based registration, the mean Euclidean displacement of corresponding optodes relative to the participant’s first session will be calculated; if it exceeds 10 mm, the cap will be repositioned and the montage redigitized before recording. The target ROI masks will be defined *a priori* in Montreal Neurological Institute space as the left and right dorsolateral prefrontal cortex (Brodmann areas 9 and 46) and the bilateral frontopolar prefrontal cortex (Brodmann area 10). The template-based candidate mapping in Supplementary Table S2 is based on the published fOLD localization of the same source–detector topology (Zimeo Morais et al., 2018; von Au et al., 2023). For each session, the proportion of each channel’s AtlasViewer-derived cortical sensitivity falling within its candidate ROI will be calculated. A channel will contribute to that ROI only when this proportion is at least 50%; otherwise, it will be excluded from ROI aggregation and will not be reassigned to another ROI in the primary analysis (de Rond et al., 2023). All anatomical eligibility decisions will be completed without reference to the fNIRS outcome data.

Before acquisition, skin tone and hair color, texture, thickness, and density will be recorded using the prespecified categorical form in Supplementary Table S3, as these characteristics may affect fNIRS signal quality (Yücel et al., 2025a). Hair beneath each optode will be separated, and optode position and pressure will be adjusted to ensure direct scalp contact. The cap and cables will then be secured to minimize changes in optode–scalp coupling.

Signal optimization will be performed in Aurora before recording. Channel quality will subsequently be assessed in non-overlapping 10-s windows using the scalp coupling index (SCI) and peak spectral power (PSP). A window will be classified as acceptable when both wavelengths contain valid, non-saturated intensity data, SCI is ≥0.80, and PSP is ≥0.10 (Hernandez and Pollonini, 2020; Yücel et al., 2021). A channel will be retained only if the quality criteria are met separately in at least 80% of the baseline windows (at least 10 of 12 windows) and at least 80% of the practice windows (at least 29 of 36 windows). The same criteria will be applied to the short-separation channels. A trial will be excluded if any prespecified ROI retains fewer than two acceptable long-separation channels. Participants must have an acceptable trial for all three practices to be included in the primary within-subject analysis.

#### 2.3.3 Physiological and motion recording

Respiratory effort and photoplethysmography (PPG) will be recorded using a NIRxWINGS2 physiological sensor module (NIRx Medical Technologies, Berlin, Germany). A respiratory belt will be placed around the lower thorax, and the PPG sensor will be secured to the non-dominant index finger, with its cable fixed along the hand and forearm to minimize traction during movement. Both signals will be sampled at 500 Hz. Head and probe motion will be recorded at 100 Hz using the NIRSport2 probe-level nine-axis inertial measurement unit, comprising a three-axis accelerometer, gyroscope, and magnetometer, positioned at Cz. All fNIRS, physiological, inertial, and event-marker signals will be acquired as a synchronized data stream in Aurora and exported within the same SNIRF dataset. Before each baseline recording, the respiratory and PPG waveforms will be visually inspected, and the sensors will be adjusted if clear respiratory or pulse cycles are not present.

Markers will identify the baseline, routine onset and offset, and the beginning of each movement unit; cue-defined vocalization periods during Liuzijue will also be marked. Respiratory, pulse, and motion signals will be used to characterize condition-related physiological and motion differences and to support the sensitivity analyses described below. These variables will not be treated solely as measurement noise because they may also represent intrinsic components of the traditional mind–body practices. Although this multimodal recording can help characterize systemic physiology and movement, end-tidal carbon dioxide and continuous blood pressure will not be recorded (Scholkmann et al., 2022; Jones et al., 2026). The respiratory belt cannot fully characterize ventilation-related changes in arterial carbon dioxide, particularly during the controlled exhalation and vocalization of Liuzijue. Residual systemic and cerebrovascular influences will therefore be considered when interpreting between-practice differences.

### 2.4 Data preprocessing

#### 2.4.1 Optical density conversion and motion artifact correction

Data preprocessing will be performed in MATLAB R2021a using Homer3 (v1.87.0), with the same prespecified processing configuration applied to all participants and practice conditions. Only channels meeting the signal-quality criteria described in Section 2.3 will enter preprocessing. Raw light-intensity data will first be converted to changes in optical density (OD).

Motion artifacts will be identified from the uncorrected OD signals using the Homer3 channel-wise detection algorithm, with tMotion = 0.5 s, tMask = 2 s, STDEVthresh = 20, and AMPthresh = 0.5 OD units (Cooper et al., 2012). The proportion of samples identified as motion-contaminated and the corresponding ENMO measures will be calculated for each trial to determine whether artifact burden differs among the three practices.

Spike-like artifacts will then be corrected using the Homer3 discrete wavelet algorithm with a Daubechies-2 wavelet and an interquartile-range parameter of 1.5 (Molavi and Dumont, 2012; Huang et al., 2022). The same procedure will be applied to all long- and short-separation channels. The number and proportion of wavelet coefficients removed will be reported for each practice. Trials will remain subject to the channel- and ROI-level exclusion criteria specified in Section 2.3. Because this procedure targets outlying wavelet coefficients associated with transient motion artifacts rather than applying additional high-pass filtering, it is intended to preserve the slow 6-min temporal trends used for the primary slope analysis.

#### 2.4.2 Filtering and hemoglobin conversion

The continuous baseline-plus-practice OD signals will be low-pass filtered at 0.09 Hz using a third-order zero-phase Butterworth filter. No high-pass filtering or polynomial detrending will be applied in the primary pipeline because the principal outcomes concern gradual changes over the 6-min practice period. Because this choice may retain very slow technical and systemic trends, prespecified sensitivity analyses based on the baseline slope, alternative detrending procedures, and slope consistency across processing stages will be conducted as described in Section 2.5.4. This approach suppresses high-frequency instrumental and physiological components while preserving the slow temporal trends of interest (Pinti et al., 2019; Yücel et al., 2021).

The filtered OD signals will be converted to changes in HbO and HbR concentration using the modified Beer–Lambert law, with fixed wavelength-specific differential pathlength factors (DPFs) of 7.25 at 760 nm and 6.38 at 850 nm. These values are commonly reported for continuous-wave fNIRS systems operating at the same wavelengths (Herold et al., 2018; Schaal et al., 2019). The same DPF values will be applied to all participants and across all three sessions.

#### 2.4.3 Short-separation regression and baseline correction

For the primary preprocessing pipeline, superficial hemodynamic components will be attenuated using a nonselective pooled strategy. For each trial, all acceptable 8-mm short-separation HbO and HbR signals will be centered, concatenated, and transformed by principal component analysis. All resulting orthogonal components will be entered with an intercept as nuisance regressors in separate ordinary least-squares models for each long-separation HbO and HbR signal (von Lühmann et al., 2020; Lemaire et al., 2026). This retains the complete short-channel signal subspace while avoiding collinearity. If no acceptable short-separation channel is available, the trial will be treated as invalid under the criteria in Section 2.3.

Recent evidence from an ecological GLM paradigm favors pooled, PCA-orthogonalized short-channel regressors over spatially restricted pairings (Lemaire et al., 2026). Because generalizability to slow movement–breathing practices and 6-min slope estimation is uncertain, sensitivity analyses will compare the primary pooled-PCA method with three alternatives: (a) the nearest acceptable short-separation channel for each long-separation channel, (b) all acceptable short-separation signals entered simultaneously without PCA, and (c) their mean signal (Gagnon et al., 2012; Wyser et al., 2020; Luke et al., 2021; An et al., 2025). If the source-adjacent short-separation channel fails quality criteria in the nearest-channel strategy, the nearest acceptable channel will be used. The direction, magnitude, and between-practice interpretation of the primary slope will be compared across all four strategies.

Respiratory-belt and PPG signals will be processed in MATLAB using prespecified scripts. The respiratory signal will be filtered with a first-order Butterworth band-pass filter from 0.01 to 2 Hz. Respiratory cycles will be detected and normalized in duration and amplitude to construct a session-specific average breath template; cycles containing clipped or flat-line samples or showing a correlation below 0.80 with the template will be rejected (Romano et al., 2023). The PPG signal will be segmented into individual pulses, and a 0–100 signal-quality index will be calculated for each pulse using the repeated-Gaussian-filter and adaptive cross-correlation procedure described by Karlen et al. (2012). A prespecified conservative threshold of 80 will be applied; pulses with a signal-quality index below 80 or containing clipped or flat-line samples will be rejected. Automated quality decisions will be visually checked using coded trial identifiers before feature extraction.

A 30-s window will be considered valid for respiratory analysis if at least 80% of its duration is represented by accepted respiratory cycles and at least two complete cycles are present; the corresponding PPG window will require at least 80% valid coverage and at least 15 accepted pulses. Trial-level physiological covariates will be calculated only when at least 19 of the 23 analysis windows are valid. Respiratory rate and heart rate will be calculated from accepted cycle intervals. Respiratory excursion amplitude will be defined as the median peak-to-trough belt amplitude and expressed as a percentage of the median amplitude during the final 30 s of the standing baseline. It will therefore be interpreted as relative respiratory excursion rather than tidal volume. Motion will be summarized using Euclidean norm minus one (ENMO), calculated as ENMO(*t*) = max(√[ax(*t*)^2^ + ay(*t*)^2^ + az(*t*)^2^] − 1 g, 0) (van Hees et al., 2013). For each trial, the mean and 6-min linear slope of respiratory rate, relative respiratory excursion amplitude, heart rate, and ENMO will be calculated from the valid 30-s windows. Trial-level and window-level covariates will be standardized separately using the pooled mean and standard deviation at the corresponding level before modeling.

Finally, the mean HbO and HbR values during the final 30 s of the 120-s standing baseline will be subtracted from the corresponding practice time series. The resulting baseline-corrected HbO and HbR signals will be used for feature extraction and statistical analysis.

The pre- and post-practice resting-state recordings will be processed separately using the same motion-artifact correction, hemoglobin conversion, and short-separation regression procedures. The corrected resting-state HbO signals will be band-pass filtered at 0.01–0.10 Hz. Functional connectivity will be estimated using pairwise Pearson correlations between the mean HbO time series of the predefined prefrontal ROIs, with the correlation coefficients converted to Fisher’s *z* values.

### 2.5 Data processing and analysis

#### 2.5.1 ROI aggregation and time-resolved feature extraction

Following preprocessing, the long-separation channels meeting both the signal-quality criteria and the prespecified anatomical eligibility criteria in Supplementary Table S2 will be averaged with equal weights to generate separate HbO and HbR time series for the left dorsolateral prefrontal cortex, right dorsolateral prefrontal cortex, and frontopolar prefrontal cortex. A global prefrontal cortex (PFC) time series will then be calculated as the unweighted mean of the three ROI-level signals. An ROI will contribute to the analysis only when it retains at least two acceptable long-separation channels, in accordance with the criteria specified in Section 2.3. The global PFC signal will be considered valid only when all three ROIs meet this requirement.

Each 6-min ROI-level and global PFC time series will be divided into 30-s windows advanced in 15-s steps, yielding 23 partially overlapping windows. For each window, three features will be calculated separately for HbO and HbR: the mean concentration change (μM), the linear slope estimated by ordinary least squares (μM/min), and the residual standard deviation after removal of the fitted linear trend (μM). The residual standard deviation will represent short-term variability that is not attributable to the within-window linear trend. A coefficient of variation will not be calculated because baseline-corrected HbO and HbR values may approach zero or take negative values.

For each practice, overall 6-min slopes will be estimated separately for the global PFC and each ROI, for both HbO and HbR, by regressing all baseline-corrected samples in the 360-s practice interval on elapsed time using ordinary least squares. Thus, the primary global PFC HbO slope will be derived directly from the complete practice time series rather than from the overlapping window summaries. Sliding-window approaches have previously been used to characterize time-varying fNIRS signals, although the optimal window length depends on the analytical objective (Li et al., 2015; Zhang and Zhu, 2020; Yücel et al., 2021). Therefore, the 30-s window and 15-s step will be fixed before analysis, and alternative window settings will be examined only in prespecified sensitivity analyses.

Exploratory stabilization will be characterized by changes in the absolute window slope and residual standard deviation over time. These measures will be treated as descriptive process features and will not be interpreted as validated steady-state indices or used to classify any window as a binary ‘steady state.’

#### 2.5.2 Primary and secondary outcomes

The primary outcome will be the overall 6-min linear slope of global PFC HbO for each practice, expressed in μM/min. The primary analysis will test whether this slope differs among Tai Chi, Baduanjin, and Liuzijue. Because the study is exploratory, no directional difference among the three practices is prespecified.

Secondary outcomes will include the global PFC HbO time-averaged concentration; the corresponding global PFC HbR slope and time-averaged concentration; ROI-specific HbO and HbR slopes and time-averaged concentrations; and the time-resolved window means, slopes, and residual standard deviations. Changes in absolute slope and residual standard deviation across windows will be treated as exploratory descriptions of hemodynamic stabilization rather than validated stabilization indices. These secondary analyses were not separately powered; effect estimates and 95% confidence intervals will therefore be emphasized, and nonsignificant results will not be interpreted as evidence of no difference.

Trial-level means and slopes of respiratory rate, relative respiratory excursion amplitude, heart rate, and ENMO, together with the proportion of samples identified as motion-contaminated and the proportion of wavelet coefficients removed, will also be summarized by practice to determine whether physiological or artifact burden differs systematically among the three conditions.

#### 2.5.3 Statistical analysis

Statistical analyses will be performed using IBM SPSS Statistics 26. Participant characteristics and data-quality measures will be summarized using means and standard deviations or medians and interquartile ranges, as appropriate, together with ranges and frequencies.

The primary outcome will be analyzed using a linear mixed-effects model estimated by restricted maximum likelihood. Practice, session period, and assigned Latin-square sequence will be included as fixed effects, and participant will be included as a random intercept. Physiological and motion measures will not be included in the primary model because they may represent intrinsic components of the traditional mind–body practices rather than measurement noise alone. The omnibus effect of practice in this model will constitute the primary statistical test. A sensitivity model will additionally include the standardized 6-min slopes of respiratory rate, relative respiratory excursion amplitude, heart rate, and ENMO. If the primary omnibus test is significant at two-sided α = 0.05, the three pairwise practice contrasts will be examined using a Bonferroni-adjusted significance level of 0.0167. Estimated mean differences, 95% confidence intervals, and exact *p* values will be reported.

The trial-level secondary hemodynamic outcomes—global PFC HbO time-averaged concentration; global PFC HbR overall slope and time-averaged concentration; and ROI-specific HbO and HbR overall slopes and time-averaged concentrations—will be analyzed using the same fixed effects and participant random intercept as the primary model. ROI-specific outcomes will be modeled separately for each prespecified ROI. Corresponding models additionally incorporating the 6-min physiological and motion slopes or trial-level physiological and motion means will be conducted as sensitivity analyses. Pairwise practice contrasts will be examined only after a significant omnibus practice effect for the corresponding outcome. Because these analyses are exploratory, the Benjamini–Hochberg procedure will control the false discovery rate at 0.05 across the pairwise contrasts within the global and ROI-specific trial-level outcome families (Benjamini and Hochberg, 1995). Adjusted mean differences, 95% confidence intervals, exact *p* values, and false-discovery-rate-adjusted q values will be reported.

The time-resolved window means, signed slopes, and residual standard deviations will be analyzed separately for HbO and HbR at the global PFC and prespecified ROI levels. Each secondary mixed-effects model will include practice, window number, the practice-by-window interaction, session period, and assigned sequence as fixed effects. Participant will be included as a random intercept, and a first-order autoregressive residual covariance structure will be used for successive windows within each participant and practice. Window number will be treated as a categorical factor so that no linear temporal trajectory is imposed. Absolute window slopes and residual standard deviations will also be examined as exploratory stabilization features. Corresponding models additionally incorporating window-level respiratory rate, relative respiratory excursion amplitude, heart rate, and ENMO will be conducted as sensitivity analyses. A significant practice-by-window interaction will be followed by pairwise practice contrasts at each window, with the Benjamini–Hochberg procedure controlling the false discovery rate at 0.05 across all window-specific contrasts for the corresponding outcome (Gueorguieva and Krystal, 2004; Tak and Ye, 2014).

Resting-state functional connectivity will be examined as an exploratory outcome using a linear mixed-effects model with practice, time (pre-practice and post-practice), the practice-by-time interaction, and session period as fixed effects and participant as a random intercept. Pre- to post-practice changes within each condition will also be examined, with adjustment for multiple comparisons.

Trial-level means and slopes of respiratory rate, relative respiratory excursion amplitude, heart rate, and ENMO will be compared among practices using the same trial-level fixed and random effects as the primary model, but without entering the corresponding measure as a covariate. Counts of motion-contaminated samples and removed wavelet coefficients will be analyzed using generalized linear mixed-effects models with a binomial distribution and logit link, with the corresponding total numbers of samples or coefficients specified as denominators. These analyses will be exploratory, and the Benjamini–Hochberg procedure will control the false discovery rate at 0.05 across pairwise practice contrasts within the physiological and artifact-burden outcome families.

Model residuals and influential observations will be examined, but the primary statistical method will not be selected according to a preliminary normality test. Regardless of the diagnostic results, a Friedman test will be conducted for the primary outcome as a prespecified nonparametric sensitivity analysis. If the omnibus Friedman test is significant at a two-sided *α* = 0.05, the three pairwise practice comparisons will be performed using paired Wilcoxon signed-rank tests with a Bonferroni-adjusted significance level of 0.0167. No participant will be excluded solely because of an extreme outcome value.

#### 2.5.4 Missing data, sensitivity analyses, and reproducibility

The primary analysis will include participants with acceptable fNIRS data for all three practices. Missing or invalid fNIRS and auxiliary-sensor data will not be imputed. The numbers and reasons for channel, trial, auxiliary-signal, and participant exclusions will be reported separately for each practice. The physiological- and motion-adjusted sensitivity analysis will include participants with valid respiratory, PPG, and motion covariates for all three practices. An available-case adjusted mixed-effects analysis including participants with at least two practice conditions with complete multimodal data will also be conducted as a sensitivity analysis.

Three prespecified sensitivity analyses will specifically evaluate the robustness of the primary global PFC HbO slope to very slow technical and systemic trends. First, the global PFC HbO slope during the full 120-s standing baseline will be calculated for each session, and the primary analysis will be repeated using a baseline-slope-adjusted outcome defined as the 6-min practice slope minus the corresponding baseline slope. Second, the primary slope will be recomputed after (a) subtraction of the linear trend estimated from the full baseline and extrapolated across the practice interval and (b) first-order polynomial detrending of the complete baseline-plus-practice record. Third, the slope will be recomputed from motion-corrected HbO before low-pass filtering, low-pass-filtered HbO before short-separation regression, and the final short-separation-regressed HbO signal. The direction, magnitude, and between-practice interpretation of the slopes will be compared across these processing stages.

Additional sensitivity analyses will compare: (1) the four short-separation regression strategies specified in Section 2.4; (2) the primary model without physiological and motion covariates versus the corresponding sensitivity model incorporating these covariates; (3) the primary 30-s/15-s window setting with 20-s/10-s and 60-s/30-s settings; (4) overlapping and non-overlapping 30-s windows; (5) results with each participant removed in turn; and (6) hemoglobin conversion using the primary wavelength-specific DPFs (7.25 at 760 nm and 6.38 at 850 nm) versus a common DPF of 6.0 at both wavelengths (Izzetoglu and Holtzer, 2020). Consistency in the direction and interpretation of the findings across these analyses will be reported rather than selecting the pipeline that produces the smallest *p* value.

The preprocessing configuration, ROI mapping, statistical analysis code, and derived-index code will be preregistered before examination of between-practice results (Schroeder et al., 2023; Schubert et al., 2025). Analysis scripts and deidentified data permitted by the participant consent and ethics approval will be made publicly available in an open repository upon publication (Yücel et al., 2025b).

## 3 Discussion

This protocol aims to explore process-level patterns of prefrontal oxygenation during Tai Chi, Baduanjin, and Liuzijue practice. Rather than relying only on pre- and post-practice assessments, this protocol investigates temporal changes in prefrontal hemodynamics during ongoing practice. This approach is important because traditional mind–body practices involve continuous coordination among movement control, breathing rhythm, attentional focus, postural stability, and intention-guided regulation (Larkey et al., 2009; Yeung et al., 2018; Shi et al., 2020; Henz and Schöllhorn, 2018). These processes may be associated with sustained changes in prefrontal hemodynamics, but their process-level prefrontal hemodynamic characteristics during practice remain insufficiently understood.

Tai Chi is characterized by continuous movement transitions, weight shifting, postural control, breath regulation, and intention-guided action, which may require sustained attentional control and movement-related cognitive demands (Larkey et al., 2009; Yeung et al., 2018; Zhang et al., 2023; Wang et al., 2025). Baduanjin includes relatively standardized guiding movements, as well as coordinated breathing and repeated postural changes, which may be associated with a temporal pattern of prefrontal oxygenation different from those observed during the other practices (Zhang and Jiang, 2023; Feng et al., 2025). Liuzijue places greater emphasis on breathing rhythm, vocalization, and attentional regulation, which may likewise be associated with a different temporal pattern of prefrontal oxygenation (Shi et al., 2020; Henz and Schöllhorn, 2018; Zhang et al., 2020). This protocol contributes to existing understanding by examining whether these different practice structures are associated with distinct trajectories of prefrontal oxygenation within the same participants. The comparison remains exploratory, and no directional differences are prespecified.

One of the major methodological features of this protocol is the use of wearable fNIRS. Compared with neuroimaging methods that impose stricter movement constraints, wearable fNIRS permits data collection during relatively natural practice conditions (Herold et al., 2018; Yücel et al., 2021). This is particularly relevant to research on traditional mind–body practices, in which the training process involves continuous movement, breathing coordination, and attentional regulation. The prespecified window means, slopes, and residual standard deviations may provide a process-based description of the temporal trajectories and short-term variability of prefrontal oxygenation during practice. Changes in absolute slope and residual standard deviation will be interpreted only as exploratory stabilization features rather than as validated steady-state indices. Previous work has used dynamic analyses to characterize time-varying fNIRS signals (Li et al., 2015). Furthermore, preprocessing and feature-extraction choices have been shown to substantially influence the results generated from fNIRS data, making transparency and process sensitivity imperative in analytical methods (Tak and Ye, 2014; Bonilauri et al., 2022). Such information may extend research on traditional mind–body practices from general outcome evaluation to a process-based description of prefrontal hemodynamics during practice.

This protocol may help characterize the prefrontal hemodynamic patterns associated with the coordination of movement–breathing–attention in traditional mind–body practices. It may also offer a means to compare other traditional mind–body practices at a process level according to their prefrontal hemodynamic patterns rather than only their health-related outcomes. From the perspective of training-process monitoring and evidence-based teaching design, the protocol may provide a methodological basis for evaluating whether wearable fNIRS can yield useful process-level indicators. Future studies using the protocol may further integrate end-tidal carbon dioxide, continuous blood pressure, and behavioral measures to improve the interpretation of prefrontal hemodynamic responses during traditional mind–body practices.

### 3.1 Anticipated limitations

There are a number of anticipated limitations of this protocol. First, the target of 36 complete datasets is based on an *a priori* power analysis for the primary omnibus comparison; however, participants will be recruited from a single university and restricted to healthy students aged 18–22 years. This homogeneous sample limits generalizability to older adults, clinical populations, and experienced practitioners—groups for whom these practices are often prescribed. Although the balanced complete-case design makes the repeated-measures ANOVA power approximation informative for the primary random-intercept LMM, the calculation does not explicitly represent the period and sequence terms or departures from compound symmetry. The time-resolved and ROI-specific analyses were not separately powered; their effect estimates and 95% confidence intervals will be interpreted cautiously, and nonsignificant findings will not be taken as evidence of no difference. Second, the protocol focuses on three slow and stable traditional mind–body practices, namely Tai Chi, Baduanjin, and Liuzijue. Thus, the findings are not necessarily applicable to other physical practices characterized by explosive or combat-related movements. Despite standardized training and competence assessment, there may be individual differences in movement familiarity, rhythm control, and breathing coordination, and these factors may influence the observed prefrontal hemodynamic patterns. Third, no time-matched non-practice control condition is included. The study can therefore compare prefrontal hemodynamic patterns among the three traditional mind–body practices but cannot determine whether within-practice temporal changes differ from those occurring during a non-practice condition. Within-practice temporal changes may therefore reflect generic effects of upright activity, time on task, breathing, or sustained attention rather than the specific movement–breathing–attention structure of a given practice. Moreover, because the three practices differ in movement structure, breathing instructions, verbal cueing, and Liuzijue vocalization, between-practice differences cannot be attributed to movement characteristics alone. Fourth, although short-separation regression and synchronized respiratory, PPG, and motion recordings are included, end-tidal carbon dioxide and continuous blood pressure will not be measured. The respiratory belt cannot fully characterize ventilation-related changes in arterial carbon dioxide, particularly during the controlled exhalation and vocalization of Liuzijue. Ventilation-induced changes in PaCO₂ can alter cerebral blood flow independently of task-related cortical processes and may therefore obscure or mimic apparent practice-specific differences, particularly during Liuzijue. Consequently, residual systemic and cerebrovascular influences cannot be fully excluded. Finally, the montage covers only the PFC, anatomical localization will use participant-specific three-dimensional optode coordinates registered to a standard adult head model rather than individual structural MRI, and fNIRS measures hemodynamic rather than direct neural electrical activity. Accordingly, future findings should be interpreted as fNIRS-derived prefrontal hemodynamic patterns that may include systemic components, rather than as direct evidence of neural activity or psychological effects. Future research should examine broader populations and levels of practice experience, include appropriate time-matched control conditions, and integrate additional cerebrovascular, behavioral, and broader cortical measures.
