# Supplementary material for: Temporal patterns of prefrontal hemodynamics during Tai Chi, Baduanjin, and Liuzijue: a wearable fNIRS study protocol
Source: Front Psychol. 2026 Oct 14;17:1924514. doi: 10.3389/fpsyg.2026.1924514 (PMC13713364; doi:10.3389/fpsyg.2026.1924514)
Supplement: Supplementary file 1 [file Table_1.docx]

**Supplementary Material**

**Supplementary Table S1. Prespecified 360-s audio-cue and practice schedule**

All three tracks use the same low-volume non-lyrical instrumental background and the same output level. Practice-specific verbal cues are time-locked to event markers. No music or verbal cue is presented during the 120-s standing baseline.

| **Practice** | **Time (s)** | **Movement unit and verbal cue** | **Breathing or vocalization instruction** |
| --- | --- | --- | --- |
| 24-form Tai Chi | 0–10 | 1. 起势 (Commencing Form); the Chinese movement name is spoken once at onset. | Natural breathing; no phase-specific breathing cue. |
| 24-form Tai Chi | 10–35 | 2. 左右野马分鬃 (Part the Wild Horse's Mane); the Chinese movement name is spoken once at onset. | Natural breathing; no phase-specific breathing cue. |
| 24-form Tai Chi | 35–47 | 3. 白鹤亮翅 (White Crane Spreads Its Wings); the Chinese movement name is spoken once at onset. | Natural breathing; no phase-specific breathing cue. |
| 24-form Tai Chi | 47–72 | 4. 左右搂膝拗步 (Brush Knee and Twist Step); the Chinese movement name is spoken once at onset. | Natural breathing; no phase-specific breathing cue. |
| 24-form Tai Chi | 72–82 | 5. 手挥琵琶 (Play the Lute); the Chinese movement name is spoken once at onset. | Natural breathing; no phase-specific breathing cue. |
| 24-form Tai Chi | 82–107 | 6. 左右倒卷肱 (Repulse Monkey); the Chinese movement name is spoken once at onset. | Natural breathing; no phase-specific breathing cue. |
| 24-form Tai Chi | 107–127 | 7. 左揽雀尾 (Grasp the Sparrow's Tail—Left); the Chinese movement name is spoken once at onset. | Natural breathing; no phase-specific breathing cue. |
| 24-form Tai Chi | 127–147 | 8. 右揽雀尾 (Grasp the Sparrow's Tail—Right); the Chinese movement name is spoken once at onset. | Natural breathing; no phase-specific breathing cue. |
| 24-form Tai Chi | 147–159 | 9. 单鞭 (Single Whip); the Chinese movement name is spoken once at onset. | Natural breathing; no phase-specific breathing cue. |
| 24-form Tai Chi | 159–184 | 10. 云手 (Wave Hands Like Clouds); the Chinese movement name is spoken once at onset. | Natural breathing; no phase-specific breathing cue. |
| 24-form Tai Chi | 184–194 | 11. 单鞭 (Single Whip); the Chinese movement name is spoken once at onset. | Natural breathing; no phase-specific breathing cue. |
| 24-form Tai Chi | 194–204 | 12. 高探马 (High Pat on Horse); the Chinese movement name is spoken once at onset. | Natural breathing; no phase-specific breathing cue. |
| 24-form Tai Chi | 204–214 | 13. 右蹬脚 (Right Heel Kick); the Chinese movement name is spoken once at onset. | Natural breathing; no phase-specific breathing cue. |
| 24-form Tai Chi | 214–226 | 14. 双峰贯耳 (Strike Ears with Both Fists); the Chinese movement name is spoken once at onset. | Natural breathing; no phase-specific breathing cue. |
| 24-form Tai Chi | 226–236 | 15. 转身左蹬脚 (Turn and Left Heel Kick); the Chinese movement name is spoken once at onset. | Natural breathing; no phase-specific breathing cue. |
| 24-form Tai Chi | 236–251 | 16. 左下势独立 (Snake Creeps Down and Golden Rooster Stands on One Leg—Left); the Chinese movement name is spoken once at onset. | Natural breathing; no phase-specific breathing cue. |
| 24-form Tai Chi | 251–266 | 17. 右下势独立 (Snake Creeps Down and Golden Rooster Stands on One Leg—Right); the Chinese movement name is spoken once at onset. | Natural breathing; no phase-specific breathing cue. |
| 24-form Tai Chi | 266–286 | 18. 左右穿梭 (Fair Lady Works at Shuttles); the Chinese movement name is spoken once at onset. | Natural breathing; no phase-specific breathing cue. |
| 24-form Tai Chi | 286–296 | 19. 海底针 (Needle at Sea Bottom); the Chinese movement name is spoken once at onset. | Natural breathing; no phase-specific breathing cue. |
| 24-form Tai Chi | 296–306 | 20. 闪通臂 (Fan Through Back); the Chinese movement name is spoken once at onset. | Natural breathing; no phase-specific breathing cue. |
| 24-form Tai Chi | 306–324 | 21. 转身搬拦捶 (Turn, Deflect Downward, Parry, and Punch); the Chinese movement name is spoken once at onset. | Natural breathing; no phase-specific breathing cue. |
| 24-form Tai Chi | 324–339 | 22. 如封似闭 (Apparent Close-Up); the Chinese movement name is spoken once at onset. | Natural breathing; no phase-specific breathing cue. |
| 24-form Tai Chi | 339–347 | 23. 十字手 (Cross Hands); the Chinese movement name is spoken once at onset. | Natural breathing; no phase-specific breathing cue. |
| 24-form Tai Chi | 347–360 | 24. 收势 (Closing Form); the Chinese movement name is spoken once at onset. | Natural breathing; no phase-specific breathing cue. |
| Baduanjin | 0–10 | 预备式 (Opening Posture); the Chinese movement name is spoken once at onset. | Natural breathing. |
| Baduanjin | 10–52 | 1. 两手托天理三焦 (Two Hands Hold up the Heavens); the Chinese movement name is spoken once at onset. | Three paced repetitions with standardized inhale and exhale cues. |
| Baduanjin | 52–94 | 2. 左右开弓似射雕 (Draw the Bow to Shoot the Hawk); the Chinese movement name is spoken once at onset. | Three paced repetitions with standardized inhale and exhale cues. |
| Baduanjin | 94–136 | 3. 调理脾胃须单举 (Raise One Hand to Regulate the Spleen and Stomach); the Chinese movement name is spoken once at onset. | Three paced repetitions with standardized inhale and exhale cues. |
| Baduanjin | 136–178 | 4. 五劳七伤往后瞧 (Look Back to Relieve Strain); the Chinese movement name is spoken once at onset. | Three paced repetitions with standardized inhale and exhale cues. |
| Baduanjin | 178–220 | 5. 摇头摆尾去心火 (Sway the Head and Shake the Tail); the Chinese movement name is spoken once at onset. | Three paced repetitions with standardized inhale and exhale cues. |
| Baduanjin | 220–262 | 6. 两手攀足固肾腰 (Two Hands Hold the Feet to Strengthen the Kidneys and Waist); the Chinese movement name is spoken once at onset. | Three paced repetitions with standardized inhale and exhale cues. |
| Baduanjin | 262–304 | 7. 攒拳怒目增气力 (Clench the Fists and Glare Fiercely); the Chinese movement name is spoken once at onset. | Three paced repetitions with standardized inhale and exhale cues. |
| Baduanjin | 304–346 | 8. 背后七颠百病消 (Rise on the Toes Seven Times); the Chinese movement name is spoken once at onset. | Three paced repetitions with standardized inhale and exhale cues. |
| Baduanjin | 346–360 | 收势 (Closing Posture); the Chinese movement name is spoken once at onset. | Natural breathing. |
| Liuzijue | 0–15 | 预备式 (Yubeishi); the Chinese unit name is spoken once at onset. | Natural breathing. |
| Liuzijue | 15–67 | 1. 嘘 (Xu); the Chinese unit name is spoken once at onset. | Four cycles: nasal inhalation followed by controlled exhalation with 嘘 (Xu). |
| Liuzijue | 67–119 | 2. 呵 (He); the Chinese unit name is spoken once at onset. | Four cycles: nasal inhalation followed by controlled exhalation with 呵 (He). |
| Liuzijue | 119–171 | 3. 呼 (Hu); the Chinese unit name is spoken once at onset. | Four cycles: nasal inhalation followed by controlled exhalation with 呼 (Hu). |
| Liuzijue | 171–223 | 4. 呬 (Si); the Chinese unit name is spoken once at onset. | Four cycles: nasal inhalation followed by controlled exhalation with 呬 (Si). |
| Liuzijue | 223–275 | 5. 吹 (Chui); the Chinese unit name is spoken once at onset. | Four cycles: nasal inhalation followed by controlled exhalation with 吹 (Chui). |
| Liuzijue | 275–327 | 6. 嘻 (Xi); the Chinese unit name is spoken once at onset. | Four cycles: nasal inhalation followed by controlled exhalation with 嘻 (Xi). |
| Liuzijue | 327–360 | 收势 (Shoushi); the Chinese unit name is spoken once at onset. | Natural breathing. |

*Note. Tai Chi and Baduanjin movement names are presented in Chinese followed by English translations in parentheses. Liuzijue sounds are presented in Chinese followed by pinyin without tone marks. All verbal movement cues in the experimental audio tracks will be delivered in Chinese. Within each Liuzijue sound segment, cycle-onset cues occur at 0, 13, 26, and 39 s relative to segment onset. The finalized audio files and corresponding event-marker files will be archived with the preregistered protocol.*

**Supplementary Table S2. Template-based candidate channel-to-region-of-interest mapping and anatomical eligibility criteria**

| **Channel** | **Source–detector pair** | **Candidate primary ROI** | **Published template-based anatomical correspondence** |
| --- | --- | --- | --- |
| Ch1 | S1–D1 | Frontopolar PFC | Left frontopolar area, 55% |
| Ch2 | S1–D2 | Frontopolar PFC | Right frontopolar area, 55% |
| Ch3 | S1–D3 | Frontopolar PFC | Frontopolar area, 88% |
| Ch4 | S2–D1 | Not assigned | Left DLPFC, 25%; left frontopolar area, 25% |
| Ch5 | S2–D4 | Not assigned | Left DLPFC, 43%; left pars triangularis, 49% |
| Ch6 | S3–D2 | Not assigned | Right frontopolar area, 31%; right DLPFC, 20% |
| Ch7 | S3–D7 | Not assigned | Right DLPFC, 43%; right pars triangularis, 44% |
| Ch8 | S4–D1 | Frontopolar PFC | Left frontopolar area, 70% |
| Ch9 | S4–D3 | Frontopolar PFC | Left frontopolar area, 76% |
| Ch10 | S4–D4 | Not assigned | Left DLPFC, 49% |
| Ch11 | S5–D2 | Frontopolar PFC | Right frontopolar area, 69% |
| Ch12 | S5–D3 | Frontopolar PFC | Right frontopolar area, 73% |
| Ch13 | S5–D7 | Not assigned | Right DLPFC, 47%; right frontopolar area, 19% |
| Ch14 | S6–D4 | Not assigned | Left DLPFC, 22%; left pars triangularis, 73% |
| Ch15 | S6–D5 | Left DLPFC | Left DLPFC, 92% across Brodmann areas 9 and 46 |
| Ch16 | S7–D3 | Not assigned to a lateralized ROI | DLPFC, 62%; frontopolar area, 20%; template MNI x-coordinate close to the midline |
| Ch17 | S7–D5 | Left DLPFC | Left DLPFC, 63% |
| Ch18 | S7–D6 | Right DLPFC | Right DLPFC, 69% |
| Ch19 | S8–D6 | Right DLPFC | Right DLPFC, 90% across Brodmann areas 9 and 46 |
| Ch20 | S8–D7 | Not assigned | Right DLPFC, 24%; right pars triangularis, 71% |

*Note. DLPFC, dorsolateral prefrontal cortex; PFC, prefrontal cortex. The candidate assignments are based on the published fOLD localization of the same source–detector topology (Zimeo Morais et al., 2018; von Au et al., 2023). Frontopolar PFC is defined as bilateral Brodmann area 10, whereas the left and right DLPFC are defined as the corresponding hemispheric portions of Brodmann areas 9 and 46. For each session, a candidate channel will contribute to its prespecified ROI only when at least 50% of its AtlasViewer-derived cortical sensitivity falls within that ROI and the channel also meets the signal-quality criteria. Channels not meeting the anatomical threshold will not be reassigned to another ROI in the primary analysis. A minimum of two eligible and acceptable channels per ROI will still be required.*

**Supplementary Table S3. Prespecified skin, hair, cap-placement, and optical-contact record**

| **Variable** | **Recording method or response options** | **Timing** | **Use in quality reporting** |
| --- | --- | --- | --- |
| Head circumference | Measured in centimeters at the standardized cap line | First session; confirm each session | Cap-size selection and descriptive reporting |
| Cap size | Manufacturer size used; same size retained across sessions | Each session | Placement reproducibility |
| Skin tone | Monk Skin Tone Scale, category 1–10, matched under standardized room lighting | First session | Participant-level signal-quality descriptor |
| Hair color | Black; dark brown; light brown; blond; red; gray/white; dyed/other | First session; update if changed | Participant-level signal-quality descriptor |
| Hair texture | Straight; wavy; curly; coily | First session | Participant-level signal-quality descriptor |
| Hair-shaft thickness | Fine; medium; coarse | First session | Participant-level signal-quality descriptor |
| Hair density at montage sites | None/shaved; sparse; moderate; dense, rated after creating the optode parting | Each session | Session-level signal-quality descriptor |
| Hair management | Optode-site hair separated: yes/no; adjustment repeated: yes/no | Each session | Documents preparation procedures |
| Direct scalp contact | Achieved for every optode: yes/no; optodes requiring refitting recorded by identifier | Each session before acquisition | Documents optical-contact optimization |
| Initial Aurora calibration | Excellent; acceptable; critical; lost, recorded for every channel | Each session before acquisition | Compared with prespecified SCI/PSP acceptance results |
| Final channel acceptance | Accepted/rejected for each wavelength and channel according to SCI ≥ 0.80, PSP ≥ 0.10, valid non-saturated intensity, and ≥80% acceptable windows separately in the baseline (≥10 of 12) and practice (≥29 of 36) periods | After preprocessing | Channel-, ROI-, trial-, and participant-flow reporting |
| 3D placement record | Coordinate file complete: yes/no; mean displacement from Session 1 (mm); repositioning/redigitization performed: yes/no | Each session | Between-session placement consistency |

*Note. SCI, scalp coupling index; PSP, peak spectral power. The skin and hair fields are prespecified metadata and will not be used to exclude participants. Their associations with signal quality and channel retention will be reported descriptively.*
